# Cerebellar damage impairs the self-rating of regret feeling in a gambling task

**DOI:** 10.3389/fnbeh.2015.00113

**Published:** 2015-05-05

**Authors:** Silvia Clausi, Giorgio Coricelli, Iolanda Pisotta, Enea Francesco Pavone, Marco Lauriola, Marco Molinari, Maria Leggio

**Affiliations:** ^1^Department of Psychology, Sapienza University of RomeRome, Italy; ^2^Ataxia Laboratory, IRCCS Santa Lucia FoundationRome, Italy; ^3^Department of Economics, University of Southern CaliforniaLos Angeles, CA, USA; ^4^Neurological and Spinal Cord Injury Rehabilitation Department A, IRCCS Santa Lucia FoundationRome, Italy; ^5^Social and Cognitive Neuroscience Lab, IRCCS Santa Lucia FoundationRome, Italy; ^6^Braintrends Ltd, Applied NeuroscienceRome, Italy; ^7^Department of Developmental and Social Psychology, Sapienza University of RomeRome, Italy

**Keywords:** choice behavior, cortico-cerebellar circuits, emotion, gambling, self-monitoring, social cognition, autism spectrum disorders, alexithymia

## Abstract

Anatomical, clinical, and neuroimaging evidence implicates the cerebellum in processing emotions and feelings. Moreover recent studies showed a cerebellar involvement in pathologies such as autism, schizophrenia and alexithymia, in which emotional processing have been found altered. However, cerebellar function in the modulation of emotional responses remains debated. In this study, emotions that are involved directly in decision-making were examined in 15 patients (six males; age range 17–60 years) affected by cerebellar damage and 15 well matched healthy controls. We used a gambling task, in which subjects’ choices and evaluation of outcomes with regard to their anticipated and actual emotional impact were analyzed. Emotions, such as regret and relief, were elicited, based on the outcome of the unselected gamble. Interestingly, despite their ability to avoid regret in subsequent choices, patients affected by cerebellar lesions were significantly impaired in evaluating the feeling of regret subjectively. These results demonstrate that the cerebellum is involved in conscious recognizing of negative feelings caused by the sense of self-responsibility for an incorrect decision.

## Introduction

Counterfactual reasoning is a cognitive mechanism that allows one to evaluate and compare what is obtained with what would have been acquired if a different choice had been made. Thus, counterfactually people will react emotionally to what has been obtained and to the alternative unobtained outcome. Ultimately, counterfactual thinking refers to the generation of alternatives to factual events and appears to be a pervasive feature of normal cognition. Specifically, counterfactual thoughts highlight causal relationships between choices and outcomes, thereby suggesting future courses of action that might be implemented strategically to facilitate adaptive behavior (Byrne, 2002). Moreover, counterfactual thinking is more related to negative emotions, regret and disappointment, than to positive emotions (Rose, 1997).

Whereas disappointment arises when a negative outcome occurs independently of our decisions, regret occurs exclusively when the outcome results from bad decisions. Thus, regret differs from disappointment, because the former is related to a strong sense of personal responsibility. The experience of regret has a powerful influence on subsequent behavioral choices—its anticipation leads to an adjustment of subsequent decisions (“regret-aversive” behavior) to minimize ensuing regrettable experiences (Zeelenberg et al., 1996; Camille et al., 2004; Coricelli et al., 2005).

Neuropsychological and neuroimaging studies have demonstrated a dissociation between disappointment and regret. Whereas disappointment correlates with activation in the middle temporal gyrus and dorsal brainstem (Coricelli et al., 2005), the orbitofrontal cortex has been shown to be an important region for the experience and anticipation of regret (Camille et al., 2004; Coricelli et al., 2005; Chua et al., 2009). For instance, patients with lesions in the orbitofrontal cortex do not report regret or anticipate it in subsequent choices (Camille et al., 2004). A reduced feeling of regret was also recently found in patients with autism spectrum disorders (Zalla et al., 2014), in which cerebellar structural and functional alterations have been largely described (Fatemi et al., 2012).

Several studies have reported cerebellar activation during tasks that involve decision-making processes (Guggisberg et al., 2008; Rosenbloom et al., 2012). The cerebellum has also been proposed to function in decision-making tasks as a detector of error signals to improve future performance (Ernst et al., 2002). These studies clearly implicate the cerebellum in decision-making processes. Although the cerebellum has been linked to the modulation of cognitive and emotional behaviors, based on its large anatomical and functional connections through the dentate nuclei with the prefrontal, temporo-parietal, and limbic areas (Schmahmann, 2010; D’Angelo and Casali, 2013), whether the cerebellum functions in integrating the emotional and cognitive components during decision-making has not been examined and could be of interest in order to clarify its role in pathologies such as schizophrenia, autism and alexithymia.

We studied the involvement of the cerebellum in feeling disappointment and regret, specifically seeking to determine whether a damage in this region impairs the ability to report regret or incorporate regret into the decision-making process to improve future choices. To this end, we implemented the regret gambling task, developed originally by Mellers et al. (1999) and adapted by Camille et al. (2004).

## Materials and Methods

### Subjects

Fifteen right-handed patients (six males), recruited at IRCCS Santa Lucia Foundation Rehabilitation Hospital and affected by cerebellar damage, participated in the study. Gender, mean age, and mean years of education are reported in Table 1.

**Table 1:** **Groups’ characteristics**.

	Gender m/f	Age mean (s.d.)	Education level mean (s.d.)	Intellectual level mean (s.d.)	ICARS: kinetic mean score (s.d.)	ICARS: total mean score (s.d.)
Patients	6/9	42.8 (10.8)	14 (3.2)	28.7 (2.3)	16.43 (11.1)	39.97 (24.04)
Controls	6/9	41.9 (11.6)	14.5 (2.9)	30.7 (1.5)	/	/

*m = males; f = females; Intellectual level was evaluated by PM47; s.d. = standard deviations; ICARS = International Co-operative Ataxia Rating Scale (Trouillas et al., 1997)*.

Patients were affected by degenerative (*N* = 10), focal unilateral (*N* = 4), and bilateral (*N* = 1) cerebellar lesions. Subjects with degenerative pathologies were affected by Friedreich ataxia (*N* = 3), spinocerebellar ataxia type 2 (SCA 2) (*N* = 3), SCA 15 (*N* = 2), SCA 28 (*N* = 1) and idiopathic cerebellar ataxia (ICA) (*N* = 1) (Table 2).

**Table 2:** **Patients’ lesion characteristics**.

Case	Lesion	Side	Etiology	DCN	ANT	POST	Hem	Vermis
B.A.	Focal	R	Surgical	x	x	x	x	x
B.F.	Atrophic	/	ICA	-	-	-	-	-
C.P.	Focal	B	Surgical	x	-	x	x	x
C.M.	Atrophic	/	Friedreich	-	-	-	-	-
R.S.	Atrophic	/	Friedreich	-	-	-	-	-
R.F.	Atrophic	/	Friedreich	-	-	-	-	-
S.S.	Focal	R	Surgical	x		x	x	-
Sm.S.	Focal	R	Surgical	x	x	x	-	x
T.S.	Atrophic	/	SCA2	-	-	-	-	-
Z.E.	Focal	R	Ischemic	x	x	x	x	x
L.I.	Atrophic	/	SCA2	-	-	-	-	-
S.C.	Atrophic	/	SCA28	-	-	-	-	-
V.D.	Atrophic	/	SCA15	-	-	-	-	-
V.C.	Atrophic	/	SCA15	-	-	-	-	-
M.V.	Atrophic	/	SCA2	-	-	-	-	-

*R = right; B = bilateral; SCA = spinocerebellar atrophy; DCN = deep cerebellar nuclei; ANT = anterior cerebellar lobe; POST = posterior cerebellar lobe; Hem = cerebellar hemisphere*.

Focal lesions resulted from ischemic stroke or surgical ablation due to arteriovenous malformations or tumors.

**Figure 1:**
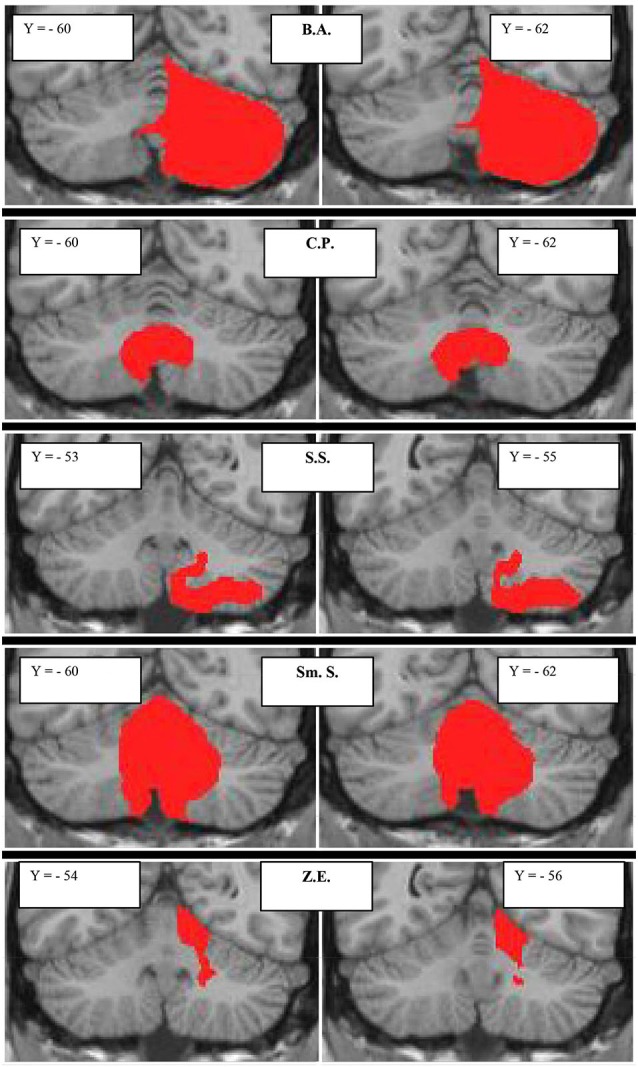
**Subjects with focal cerebellar lesions**. Lesion extensions were assessed on 3D-T1-MPRAGEs after spatial normalization and overlaid onto a coronal T1-weighted template from Schmahmann et al. (2000). For each subject, the lesion is shown in two representative coronal sections. The case code is as in Table 2.

The lesion characteristics of patients with focal damage, according to the MRI images, are described in Figure 1 and Table 2.

All patients underwent a neurological examination, and their motor impairment was quantified using the International Cooperative Ataxia Rating Scale (ICARS; Trouillas et al., 1997), which ranges from 0 (absence of any deficit) to 100 (presence of all deficits to the highest degree). Subscale score for the kinetic limbs range from 0 to 52 (Table 1).

Fifteen subjects without a history of neurological or psychiatric illness who were recruited from a pool of patients’ relatives and volunteers formed the healthy control group. Controls were matched for gender, age, education, and intellectual level (assessed by Raven’s 47 Progressive Matrices) (PM47; Raven, 1947; Table 1).

The experimental procedures were approved by the ethical committee of IRCCS Santa Lucia Foundation (CE-PROG.2-AG4-187), and written consent was obtained from each subject per the Helsinki Declaration.

### Neuropsychological Assessment

The patients’ general cognitive profiles were assessed using subtests of the Mental Deterioration Battery (BDM; Caltagirone et al., 1995). Specifically, we considered PM47 to be a measure of general cognitive ability. Praxis was measured by freehand copying of a drawing and drawing with landmarks (Gainotti et al., 1977). Verbal memory and visuospatial working memory (Carlesimo et al., 1996) were evaluated through digit span, the Corsi test (Corsi, 1972), and an immediate visual recognition task. FAS verbal fluency was used to measure verbal production (Borkowsky et al., 1967). Sentence comprehension and executive functions were examined with the Token test and the Wisconsin card sorting test, respectively (De Renzi and Vignolo, 1962; Heaton et al., 2006). Visual exploration was evaluated by barrage tasks (line cancellation task and double line cancellation tasks) (Albert, 1973; Zazzo, 1980).

### Decision-Making Tasks

The decision-making behavior of patients and control subjects was assessed using the counterfactual inference test and the gambling task.

### Counterfactual Inference Test

The Counterfactual Inference Test (CIT; Hooker et al., 2000) was used to measure the counterfactual reasoning. The CIT is a questionnaire that comprises 4 forced-choice questions. This test assumes that counterfactuals are more pronounced when the relationship between previous actions and the outcome is unusual or when there is increased physical and temporal proximity between the alternative situations.

Some example questions are: (i) “Ann gets sick after eating at a restaurant that she often visits. Sarah gets sick after eating at a restaurant that she has never visited before. Who is more upset about her choice of restaurant?” (ii) “Ed is attacked by a mugger only 10 feet from his house. James is attacked by a mugger 1 mile from his house. Who is more upset by the mugging?”

In a normal population, the target responses are Sarah for the first item and Ed for the second. The scale ranges from 0 (no counterfactual thinking) to 4 (perfect ability with regard to counterfactual thinking). For each item, events that are experienced by two individuals are presented, and three possible responses are given. Correct and incorrect responses are scored 1 and 0, respectively.

### Gambling Task

**Figure 2:**
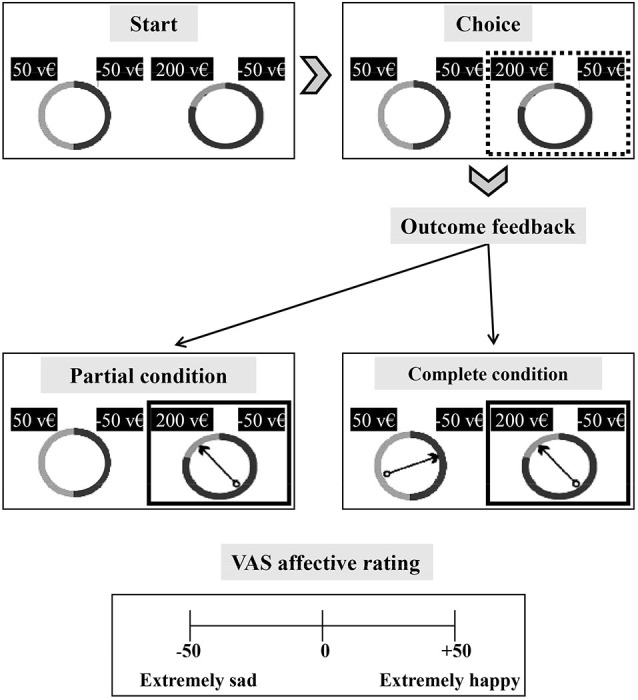
**Schematic of the regret gambling task**. Each subject was required to choose the more advantageous alternative between two wheels of fortune shown on a monitor. Each wheel (upper part of the figure) has 2 sectors associated with different value pairs. Possible outcomes are formed by any pair of combinations of the following values: 50, −50, 200, −200 (virtual money—v€) and are indicated above the wheel. Each value is randomly associated with one of three outcome probabilities (0.8, 0.2, 0.5). The length of each segment (gray and black segments) indicates the outcome probability. In the choice phase, the subject selects one of two wheels, and a rectangular box appears around the selected wheel (upper right of the figure). In the outcome phase of the partial condition blocks, a spinning arrow appears only in the selected wheel and stops in 1 of the 2 segments. Only the outcome of the selected wheel can be seen (middle left of figure). In the outcome phase of the complete condition blocks, a spinning arrow appears in both the selected and unselected wheels. The arrows rotate and stop, allowing the subject to view the outcomes of both wheels (middle right of figure). At the end of each trial, the subject has to rate his affective state by visual analogical scale (VAS) rating from –50 (extremely sad) to 50 (extremely happy) (lower part of the figure).

We adopted the gambling task that has been used by Camille et al. (2004). The task was projected onto a computer monitor. In each trial, subjects chose between two bets, on two “wheels of fortune”. The wheels had 2 segments (gray and black) that were randomly associated with various probabilities (0.2, 0.5, and 0.8) of winning or losing a specific amount of virtual money (200, 50, −50, −200). The length of each segment was proportional to the probability of an outcome (Figure 2).

At the start of the trial, the participant chose one of the two wheels (left or right) by using the keyboard (the subject had to press the key “V” and “N” respectively). Then, the bet was highlighted by a white square, and an arrow appeared in the center of the gamble and began to rotate. Two conditions were run: (i) partial; and (ii) complete. In the partial condition, the spinning arrow appeared only in the selected wheel, rotated for varying durations, and stopped in one of the two sectors. Only the outcome of the selected wheel was shown. In this condition, the subject could only evaluate the obtained and unobtained outcomes of the bet.

In the complete condition, rotating arrows appeared in the selected and unselected wheels, and when they stopped (simultaneously), they indicated the outcomes of the bet and the unchosen bet. Thus, complete feedback trials allowed the subjects to compare the outcome that was obtained in the selected gamble with that of the unchosen wheel and determine the financial consequence of their decision with regard to the advantageous and disadvantageous choice. The experimental design included a practice session of 5 trials that covered both conditions (partial and complete). Partial and complete conditions were counterbalanced across subjects.

At the end of each trial, subjects rated their affective states on a scale from −50 (extremely sad) to +50 (extremely happy). The affective states were joy (from 0 to +50) and disappointment (from 0 to −50) for the partial condition and relief (from 0 to +50) and regret (from 0 to −50) for the complete condition. The subject used the mouse to move a cursor along the rating line.

To analyze the main components of the subjects’ choice behavior, the following parameters were considered per Camille et al. (2004) and Coricelli et al. (2005):

- emotional self-evaluation, represented by self-reported emotional evaluation that indicates the valence and intensity of the affective response to the outcome of their choices;
- choice behavior, which refers to the tendency to minimize negative emotions and maximize final outcomes. We measured (a) the minimization of future disappointment, wherein a subject chose a bet that minimized the difference between the lowest and highest outcomes, weighted by the probability of the worst possible outcome; (b) anticipation of future regret, in which a subject minimized the discrepancy between the obtained and unobtained outcomes across the two bets; and (c) maximization of expected values, in which a subject computed the sum of the outcomes of each bet, weighted by their probabilities, and chose the bet with the highest expected value.
- net earnings that were realized at the end of the partial or complete condition.

### Skin Conductance Response

Simultaneously the gambling task, the skin conductance response (SCR) was recorded in a group of 5 patients (age: mean = 49.8; s.d. = 5.6; education level: mean = 16.2; s.d. = 2.5) and 5 controls (age: mean = 47.4; s.d. = 9.2; education level: mean = 16.6; s.d. = 2.2). The SCR recording allows us to have a physiological index of the autonomic emotional reactivity (Dawson et al., 2007). AD-Instruments PowerLab 8/35 and ML116 GSR Amplifier (providing low constant-voltage AC excitation (22 mVrms @ 75 Hz)) devices were used as signal amplifier with specific GSR sensors consisting of two bipolar finger electrodes. The sensors were applied on the distal phalanx of the index and middle fingers of the left hand. The signal was sampled at 1 KHz and recorded using the software LabChart 7 (AD-Instruments, Inc.).

### Data Analysis

The analysis was conducted with the statistical software package Stata, Stata Corp, College Station, TX. Nonparametric tests were applied on the behavioral data sets.

Specifically, the Mann-Whitney test was used to appraise the statistical significance between-group differences: (a) on the CIT questionnaire score that explored counterfactual reasoning; and (b) on the net gains that were realized by patients and controls in the partial and complete feedback conditions. To examine the “self-evaluation of the emotional component” the mean scores obtained on the emotional rating scale by each subject for each affective state were analyzed by the Mann-Whitney *U* test and the Wilcoxon sign rank test to evaluate between-group and within-group differences, respectively.

Following Camille et al. (2004) and Coricelli et al. (2005), the effects of anticipated emotions on the performance in the regret gambling task were assessed by panel logit regression, in which each participants binary scores across repeated gambling trials were predicted by trial characteristics (i.e., minimization of future disappointment, anticipation of future regret and maximization of options expected value) controlling for each participant intra-individual variability.

Finally, Spearman rank order correlations were used to assess the statistical associations of emotional self-evaluation ratings with the net earnings in the complete feedback condition.

Skin conductance signal of each participant was band pass filtered using a 2 Hz low-pass and 0.05 Hz high-pass filter. For each trial, the difference between the maximum value detected in a 5-s post-stimulus time window and the baseline calculated as the average value of a 0.3 s pre-stimulus time window was computed (Romano et al., 2014). Before carrying out any analyses on SCR amplitudes, the dataset was tested for normality with the Shapiro-Wilk test. The data were normally distributed (Shapiro-Wilk test all conditions: *p* > 0.1). Thus, parametrical analysis including a mixed two way-repeated-measures ANOVA was used using Feedback (Partial vs. Complete) and Affective State (Disappointment/Joy and Regret/Relief) as within factors and Group (Controls vs. Patients) as between factor. When appropriate, the analyses were corrected by Duncan *post hoc* test.

## Results

### General Neuropsychological Assessment

Cerebellar patients showed no deficits on the general neuropsychological assessment. Their scores on this assessment (Table 3) and on Raven’s PM47 (Table 1) were within the normal range.

**Table 3:** **Patients’ neuropsychological profile**.

Tasks	Mean (s.d.)	Cut-off
**Memory**
Digit span forward	6.1 (1.4)	7 ± 2
Digit span backward	4.8 (1.2)	5 ± 2
Spatial forward	7.7 (1)	7 ± 2
Spatial backward	4.5 (0.9)	5 ± 2
Immediate Visual Memory	19.7 (0.6)	13.8
**Visuospatial analysis**
Simple Barrage (errors)	0.07 (0.3)	/
Double Barrage (errors)	0.8 (0‥9)	/
**Constructional praxis**
Copying drawings with land.	65.7 (4.8)	61.8
Copying drawings	10.1 (2‥2)	7.1
**Language**
FAS	29.7 (11)	17.3
Token test	32.4 (1.8)	32
**Executive functions**
WCST: Total errors score	50°–70°	<10°

*Patients performances are expressed in corrected scores; the performances obtained in Wisconsin Card Sorting Test (WCST) are expressed in percentile of raw score*.

### Counterfactual Inference Test

The performance of cerebellar patients on the CIT questionnaire did not differ from that of control subjects when asked to draw inferences about hypothetical social events (Mann-Whitney: *Z* = 0.08; *p* = 0.93). Thus, the cerebellar damage in our patients cohort does not affect the ability to generate alternative mental representations of past events with respect to the course of actual facts.

### Gambling task

#### Self-Evaluation of Emotional Component

The explicit processing of the psychological component, associated with the outcome of the choice, was analyzed through self-evaluation of the affective state, as measured using an emotional rating scale that was administered at the end of each trial. In the partial and complete feedback conditions, the participants expressed their degree of emotional experience (happiness/dissatisfaction) with regard to the outcome of their choice.

The partial condition triggered the emotional responses of disappointment or joy. In the partial feedback condition, as expected, all subjects expressed the positive emotion of joy when the obtained outcome was higher than the unobtained outcome of the bet and the negative emotion of disappointment when the unobtained outcome was more advantageous than the obtained outcome.

**Figure 3:**
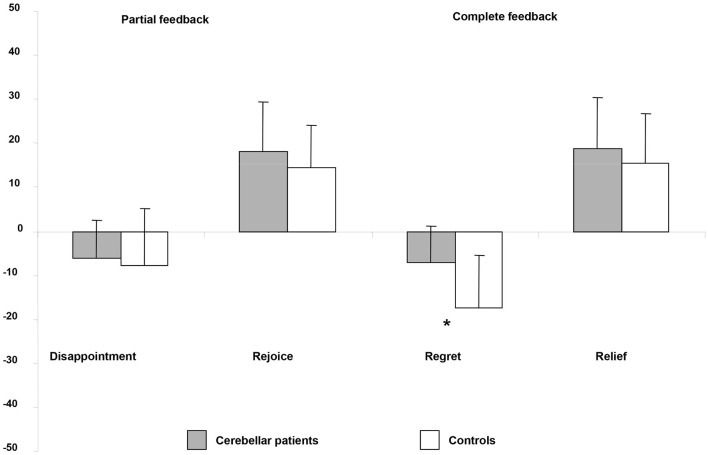
**Emotional rating, expressed as mean score of self-evaluation of the outcome of past choices in partial feedback and complete feedback conditions**. * *p* < 0.05.

No significant differences emerged in joy or disappointment between patients and controls (joy: Mann-Whitney *Z* = −1.14; *p* = 0.25; disappointment: Mann-Whitney *Z* = − 0.85; *p* = 0.39) (Figure 3).

In the complete feedback condition, when the obtained outcome was more advantageous than the outcome of the unchosen gamble, the affective rating was always positive. Patients and control subjects expressed relief, which did not differ between groups (Mann-Whitney: *Z* = −0.77; *p* = 0.44). In contrast, compared with controls, cerebellar patients experienced significantly less regret when the outcome of the selected bet was lower than that of the unchosen gamble (Mann-Whitney: *Z* = −2.21; *p* = 0.027) (Figure 3).

Furthermore, within groups comparisons did not show any significant difference between the means scores of regret and disappointment in the cerebellar patients (Wilcoxon sign rank test: *Z* = 0.39; *p* = 0.69). Conversely, the control subjects report a significantly higher level of regret than of disappointment (Wilcoxon sign rank test: *Z* = −2.61; *p* = 0.009).

These results indicate that cerebellar patients were unable to explicit their negative feeling when they are required to self-evaluate the outcome of their choice in the regret condition. Moreover, unlike control subjects the patients did not report the negative emotional feeling to a regret-inducing event as more intense than that of a disappointment-inducing event.

#### Choice Behavior

To determine whether the choices made by patients with cerebellar lesions were guided by disappointment, regret, or expected values, we adopted a choice model framework that has been used for this gambling task (Camille et al., 2004). Primarily by using this model, we examined whether patients chose to avoid regret. This analysis was performed exclusively on the data from the complete condition, which was the only condition to provide information on the outcome of the unchosen gamble that could have elicited feelings of regret (Camille et al., 2004; Coricelli et al., 2005). The subjects’ choice behavior was evaluated with respect to minimizing disappointment and regret and maximization of expected values (Table 4).

**Table 4:** **Regression analysis on subjects’ choice behavior**.

Variable		Coefficient	Standard error	*Z*	*P*
Constant	CB	0.100	0.134	0.74	0.46
	C	0.403	0.161	2.5	0.012
Disappointment	CB	−0.0004	0.002	−0.26	0.79
	C	−0.0002	0.002	−0.14	0.89
Regret	CB	0.003	0.001	2.62	0.009
	C	0.006	0.001	4.48	0.0001
Expected value	CB	0.0189	0.002	6.35	0.0001
	C	0.027	0.001	7.19	0.0001

*Regression analysis (panel logit procedure with individual random effect) on patients’ and controls’ choice behavior as a function of anticipated disappointment, anticipated regret and maximization of expected value in the “complete” condition. Patients (CB): number of subjects = 15; number of observations = 428 (two patients did not complete the task); Log likelihood = −176.33375; Wald $\chi_{(3)}^{2}$ = 115.7; Prob > χ^2^ = 0.00001. The dependent variable “choice” is equal to 1 if subject chose gamble one and is equal to 0 if subject chose gamble two. Controls (C): number of subjects = 15; number of observations = 450; Log likelihood = −136.80278; Wald $\chi_{(3)}^{2}$ = 115.01; Prob > χ^2^ = 0.00001. The dependent variable “choice” is equal to 1 if subject chose gamble one and is equal to 0 if subject chose gamble two*.

By regression analysis, patients chose by maximizing the expected value (*p* < 0.001) and minimizing future regret (*p* < 0.01) (Table 4). The data on total earnings confirmed that patients and healthy controls did not differ in terms of choice behavior.

In each condition of the gambling task, with regard to earnings, the patients made a net gain, which did not differ from the gain in the control group (partial condition: Mann-Whitney’s *U* = 105; *Z* = 0.31; *p* = 0.75; complete condition: Mann-Whitney’s *U* = 99.5; *Z* = −0.54; *p* = 0.59). We did not observe any correlation between the emotional evaluation rating and the net earnings in patients (regret: *r* = 0.40; *p* = 0.13; relief: *r* = 0.22; *p* = 0.41) or controls (regret: *r* = −0.05; *p* = 0.85; relief: *r* = 0.04; *p* = 0.86) in the complete feedback condition.

#### Skin Conductance Responses

**Figure 4:**
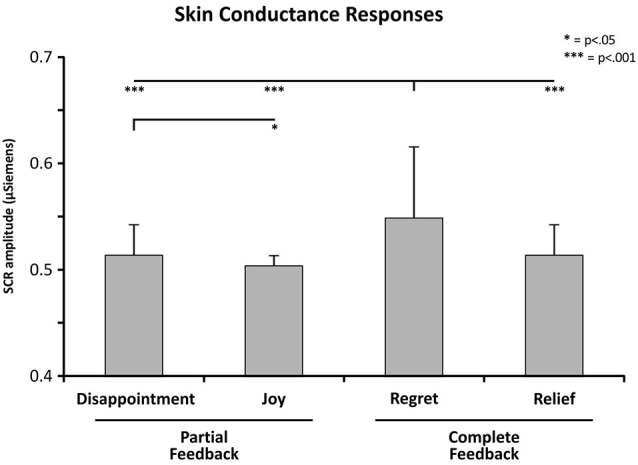
**Skin conductance response (SCR) means as a function of Feedback (partial vs. complete) and Affective State (disappointment/ joy and regret/relief) factors**. *P*-values correspond to Duncan test correction for the significant effects. Error bars indicate standard deviation of the mean.

Statistical analysis revealed (Figure 4) that the SCRs were significantly modulated by the within interaction between Feedback and Affective State factors (*F*_(1,8)_ = 9.991, *p* = 0.013; *p*^2^ = 0.55; power = 0.790). Within-group comparisons revealed higher amplitude of SCR in the Regret condition (mean in μSiemens ± s.d.; 0.55 ± 0.07) respect to Disappointment (0.51 ± 0.02; *p* < 0.001), Joy (0.50 ± 0.03; *p* < 0.001), and Relief condition (0.51 ± 0.03, *p* < 0.001). Comparison evidenced also a significant difference between Disappointment and Joy condition (*p* = 0.036). No group main effect, interactions or other comparisons were significant (*p* > 0.1).

These results indicate that the cerebellar patients showed an autonomic emotional reactivity comparable with control group. In both groups the SCRs were significantly higher in the regret condition than in the other conditions.

## Discussion

Our data demonstrate that cerebellar damage impairs the self-rating of feeling of regret. Regret is an emotion that is associated with a decision that turns out badly. It embodies the concept of liability for one’s incorrect past voluntary decision. Thus, the experience of regret has a significant influence on subsequent behavioral choices, culminating in the anticipation of this feeling to avoid further negative experiences.

This process has been examined using the gambling task paradigm used in Camille et al. (2004) in which subjects are required to choose between two gambles, each with different probabilities and expected outcomes. Regret is elicited by providing information on the outcome of the unchosen gamble (Coricelli et al., 2005). In our analysis, we focused on 3 parameters: choice behavior, self-rating of the outcome of choice, and autonomic response (i.e., SCR). Cerebellar patients and healthy controls developed similar patterns of choice behavior—i.e., they anticipated regret and maximized expected values. This result indicates that subjects with cerebellar lesions, unlike those with OFC damage (Camille et al., 2004), do not fail to anticipate the possible consequences of their choices and thus learn to avoid choices that predict future regret correctly. The integrity of the cognitive component of the choice behavior was confirmed by their performance on the CIT. Cerebellar patients had normal CIT scores, demonstrating that their capacity to generate inferences by counterfactual thinking properly was intact.

Moreover the SCR analyses evidenced that the autonomic response in cerebellar patients were comparable to that of controls in the different conditions of the gambling task.

Different studies in animals debated the role of the cerebellum in autonomic response to fear conditioning, hypothesizing that lesions of the cerebellar vermis may affect fear memory without altering baseline motor/autonomic responses to the frightening stimuli (Supple and Leaton, 1990; Sacchetti et al., 2009).

Autonomic responses are considered an index of the activation of the somatic state that is caused by the emotional experience. A normal autonomic reactivity was also described during the anticipation of disadvantageous choices in a patient affected by left cerebellar stroke (Annoni et al., 2003).

In the present study, although choice strategy and regret anticipation were preserved, the cerebellar patients experienced a significant impairment to regret self-rating. Indeed, although the cerebellar patients showed a higher arousal for regret compared to the other emotional events, their conscious perception of this negative emotion was lower than that of controls. Judgment of other feelings did not differ between groups.

These data are in line with recent evidences from rodents that support a cerebellar role in learned fear (Sacchetti et al., 2009) as demonstrated by amnesic effects of tetrodotoxin injected into the vermis after a repeatedly paired conditioned stimulus with a noxious unconditioned stimulus (Sacchetti et al., 2002).

Furthermore, interference with cerebellar activity by transcranial direct current stimulation affects the processing of negative but not positive emotions (Ferrucci et al., 2012; Ferrucci and Priori, 2014).

Overall, in the present study cerebellar patients made their choices trying to minimize the future regret; however they were unable to self-monitor it correctly.

Coricelli et al. (2005) associated the experience of regret with a neural network that comprised the medial orbitofrontal cortex (mOFC), dorsal anterior cingulate cortex, and hippocampus. This group also demonstrated the function of the dorsolateral prefrontal cortex, lateral OFC, and parietal cortex in the anticipation of regret at the time that a choice was made.

Our study presents the first evidence that the cerebellum has a role in the experience of regret. Notably, subjects with cerebellar damage were not impaired with regard to regret-based choice behavior. Conversely, cerebellar processing specifically affected the self-monitoring of regret. In estimating regret in our task, the obtained outcome (the factual, internal state) must be compared with that of the foregone choice (i.e., its counterfactual alternative, external event).

**Figure 5:**
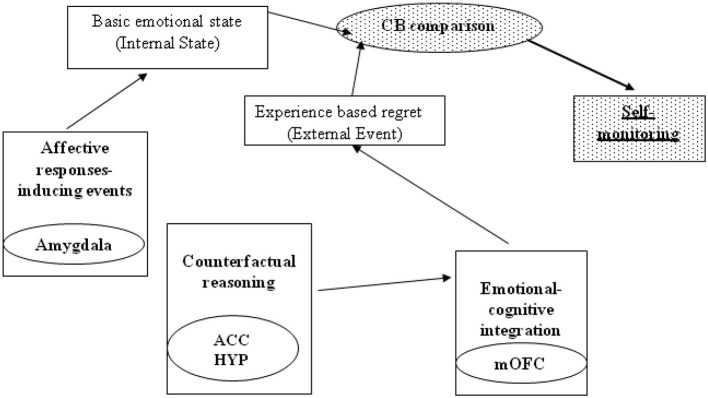
**Per Coricelli et al. (2007), the circuitry of regret comprises the medial orbitofrontal cortex (mOFC), anterior cingulated cortex (ACC), and hippocampus (Hyp)**. Regret-related activity in the ACC corresponds to the dorsal component of the ACC and thus its cognitive division. Hippocampal activity is consistent with the declarative component of regret. The interplay of the ACC and Hyp, in addition to the OFC, suggests that regret is elicited through a cognitive (top-down modulation) and declarative process. In addition of these structures, the amygdale is a critical component of a brain circuit involved in the appraisal of self-relevant events that include social stimuli (Zalla and Sperduti, 2013). Based on our data, we hypothesize that the cerebellum (CB) functions in this neural circuit. In estimating regret, the subject must compare the basic emotional state (internal state) and the state determined by the external event, based on the gambling result (external event). Considering the cerebellar involvement in estimating the state (Molinari et al., 2009), we propose that cerebellar processing intervenes specifically in the internal state vs. external event comparison.

Comparing disparate states—motor or cognitive—has always been considered the fundamental task of cerebellar function (Molinari et al., 2009). Consistent with the literature on estimating states, our findings support the model in which the cerebellum has a significant function in monitoring changes in one’s emotional state to represent negative emotions. This model is described in depth in Figure 5.

Taking into account the etiological heterogeneity of the study population, it will be interesting to analyze homogenous cohorts of patients affected by focal cerebellar lesions in order to identify whether specific cerebellar areas are involved in the proposed model.

In our study, only self-rating regret was affected in patients; self-rating of all other feelings were preserved. Regret is the only feeling that arouses a sense of personal responsibility (Camille et al., 2004; Coricelli et al., 2005; Larquet et al., 2010), which implicates self-attribution in feeling regret but not in other positive and negative affective states that arise from decision-making behavior. Relief, joy, and disappointment—but not regret—even if produced by counterfactual reasoning, lead a subject to believe that the result of his past decision is attributable to external events.

According to this hypothesis, the lateral cerebellar hemisphere shows enhanced activity in social situations implying the internal attribution of negative feelings. Cerebellar involvement is not evident when the causes of negative social events are external (e.g., other people, situational conditions) (Blackwood et al., 2003). Blackwood et al. (2003) proposed a model of processing affective states, based on *self-*agency and *other-*agency mechanisms. “Agency” refers to the sense of ownership of actions or thoughts and is central to *self*-consciousness (Gallagher, 2000). *Self*-agency implies the perception of *self*-responsibility, whereas *other*-agency implies the perception of *other*-responsibility. Notably, *other*-responsibility has been associated with disappointment (Frijda et al., 1989; Van Dijk et al., 1999), and *self*-agency and *self*-responsibility have been linked strictly to regret. In this conceptual framework, the specificity of the cerebellar influence on the self-rating of regret and the sparing of self-rating of other feelings (disappointment, joy, relief) can be interpreted, considering the cerebellar component of biological substrate for processing* self*-responsibility.

Impaired mentalization of the self, as hypothesized here, has been observed in psychiatric pathologies (Ito, 2006, 2008; D’Angelo and Casali, 2013). Certain symptoms of schizophrenia have been linked to the failure to compare internal and external representations. As in depression, psychotic symptoms have been regarded to be a loss of internal coherence between internally and externally generated signals, with a consequent dysregulation of mood homeostasis (D’Angelo and Casali, 2013). Cerebellar dysfunction has been described in these diseases, and the hypothesis that cerebellar processing might ultimately take part in generating coherent representations of the world has been advanced (Blakemore et al., 2000; Molinari et al., 2009).

According to this hypothesis, very recently by administering the same paradigm of gambling task it has been demonstrated that patients with high-functioning autism disclose a cognitive and emotional behavior resembling that of patients affected by cerebellar lesions (Zalla et al., 2014). Indeed, they show reduced regret and no difference between regret and disappointment, along with preserved counterfactual thinking and choice behavior. The authors stated that “the experience of regret crucially depends on the level of subjective responsibility and on the personal sense of blame induced by the individual’s own choice” and their data are “in accordance with research in social cognition showing the existence of unconscious guidance systems, composed by a variety of automatic process detecting relevant stimuli and information in the social and physical environments” (Zalla et al., 2014).

Taking into account that the cerebellum has been implicated in autism spectrum disorders (ASDs; Fatemi et al., 2012) with post-mortem studies showing cerebellar Purkinje cells loss (Amaral et al., 2008) and neuroimaging data showing specific cerebellar gray and white matter alterations in ASDs patients (Scott et al., 2009), the present study is the first one to demonstrates that the cerebellum has a central role in specific components of social cognition.

According to this statement, Zalla et al.’s data are consistent with evidence showing that alexithymia, considerably overlapped with ASDs, is characterized by difficulties in overtly use feeling to guide behavior. It is worth noting that both in ASDs and alexithymia the impairment of amygdala is crucial (Zalla and Sperduti, 2013; Laricchiuta et al., 2014) and, in particular, alexithymia scores have been linked directly with cerebellar areas and inversely with amygdale volume and, more in general, with limbic and para-limbic system (Laricchiuta et al., 2014).

All in all, it is reasonable to hypothesize that the cerebellum is involved in behaviors related to not-cognitively processing emotions.

## Conclusion

The present study demonstrate for the first time the involvement of the cerebellum in the self-rating of regret feelings.

This finding allows to hypothesize that the cerebellum, which is deeply interconnected with the prefrontal cortex, limbic system, and basal ganglia (Schutter and van Honk, 2005; Habas et al., 2009), has a role in the processing of feelings that are linked to the representation of the self and thus it might be involved in social cognition processing.

A limit of the present study is the heterogeneity of the subjects. Further investigation including an increasing sample size and studying specific cerebellar cohorts may contribute to better define the involvement of specific cerebellar portions within the neural circuit of regret.

## Conflict of Interest Statement

The authors declare that the research was conducted in the absence of any commercial or financial relationships that could be construed as a potential conflict of interest.
